# Human mucosal tissue-resident memory T cells in health and disease

**DOI:** 10.1038/s41385-021-00467-7

**Published:** 2021-11-06

**Authors:** Joshua Lange, Olga Rivera-Ballesteros, Marcus Buggert

**Affiliations:** Center for Infectious Medicine, Department of Medicine Huddinge, Karolinska Institutet, Stockholm, Sweden

## Abstract

Memory T cells are fundamental to maintain immune surveillance of the human body. During the past decade, it has become apparent that non-recirculating resident memory T cells (TRMs) form a first line memory response in tissues to tackle re-infections. The fact that TRMs are essential for local immunity highlights the therapeutic potential of targeting this population against tumors and infections. However, similar to other immune subsets, TRMs are heterogenous and may form distinct effector populations with unique functions at diverse tissue sites. Further insight into the mechanisms of how TRM function and respond to pathogens and malignancies at different mucosal sites will help to shape future vaccine and immunotherapeutic approaches. Here, we review the current understanding of TRM function and biology at four major mucosal sites: gastrointestinal tract, lung, head and neck, as well as female reproductive tract. We also summarize our current knowledge of how TRM targets invading pathogens and developing tumor cells at these mucosal sites and contemplate how TRMs may be exploited to protect from infections and cancer.

## Introduction

The human body, and in particular mucosal surfaces, are constantly exposed to a wide-range of pathogens and developing malignancies that the immune system needs to keep in check. The adaptive immune system serves here a critical role to mediate a memory response that rapidly could re-encounter pathogens and malignancies to limit disease propagation. In this regard, memory T cells are utterly pivotal and are mainly categorized into two different subsets with distinct functions. Memory CD4 + T cells surveil peripheral tissues for extracellular and intracellular pathogens and provide essential helper functions that augment other arms of the immune system. Memory CD8 + T cells are primarily known for their abilities to eradicate neoplastic cells and intracellular pathogens through direct lysis via cytotoxic mechanisms. As such, both memory CD4 + and CD8 + T cells coordinate immunity at mucosal surfaces to rapidly promote disease protection.

We know today that many T cells continuously migrate between tissues and blood. Early work in rats showed that T cells migrate from non-lymphoid tissues (NLTs) and secondary lymphoid tissues (SLTs) to the blood in a unidirectional manner mainly via the big lymphatic vessel thoracic duct^1–3^. A series of elegant studies in rats and sheep between the 1960s and the 1980s reported that most cells egressing from tissues are lymphocytes and mainly consist of T cells^1,4–6^. Much or our current usage of memory T cell classifications have been ramified from this early work. For instance, we still commonly separate memory T cells into central memory T cell (TCM) and effector memory T cell (TEM) subsets based on their differential capacities to home to SLTs and NLTs, respectively^7^. Also, CD4 + T cells are commonly classified using the TEM and TCM nomenclature, but these T cells are generally more plastic and are therefore commonly subdivided based on their functions (i.e., regulatory T cells, T helper 1 (Th1), Th2, Th17 and T follicular helper (Tfh) cells). However, many of these classifications are binary in nature and simplified—of obvious reason to not complicate memory classifications too much—and based on intravascular T cell phenotypes and functions. In reality, we know today from both studies in murine models and humans that circulating TEM cells can be divided into two distinct subsets: (i) cytotoxic blood-confined CX3CR1 + TEM cells and (ii) early-differentiated CX3CR1- TEM with certain stem-like properties^8–10^. These studies demonstrate a greater complexity of memory CD8 + T cell differentiation in the blood than caught through the common TCM and TEM model.

The complexity of classifying T cells based on blood is just the tip of the iceberg and do not take into consideration the fact that certain memory T cells do not leave tissues and circulate to blood. From seminal work for more than a decade ago, it became apparent that many memory T cells in NLTs are not re-circulating TEM cells, but instead stable resident populations^11–14^. These T cells are now known as tissue-resident memory T cells (TRMs) and form sentinel CD4 + and CD8 + T cell subsets in most tissues (reviewed in^15^). We now believe that residency typifies tissues for most immune lineages, where TRMs specifically dominate the total pool of virus-specific CD8 + T cells^16^ and their unique positioning within tissues allows them to generate immediate site-specific effector responses after secondary challenge to promote rapid protection^11^.

**Fig. 1:**
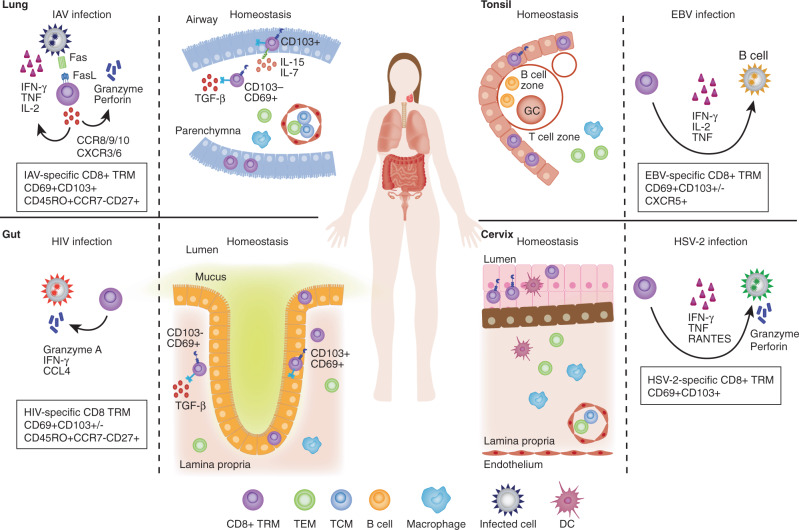
CD8 + TRMs in infections. TRMs are found in mucosal tissues following different infections. They are known to express variable levels of the tissue retention markers CD69 and CD103. Following IAV infection CD8 + TRMs infiltrate the lung epithelium and induce cell death in the targeted cells through perforin/granzyme delivery as well asl FasL/Fas pathways and produce IFNγ, TNF, IL-2, and other cytokines and chemokines to enhance inflammation and immune activation in the infected lung tissue. HIV-specific CD8 + TRMs express CD69 and intermediate levels of CD103 in the gut mucosal epithelium, where they secrete granzyme A, IFNγ and TNF against HIV-infected cells. In the tonsil, EBV-specific CD8 + TRMs localize at the lymphoepithelial barrier, where most EBV + B cells are found. Tonsillar EBV-specific CD8 + TRMs are polyfunctional and produce IFN-γ among other cytokines. After intravaginal infections HSV-specific CD8 + TRMs are established in the lower FRT, where they persist in the dermo-epidermal junction and rely on perforin/granzyme and cytokine secretion (mostly IFNγ, but also TNF and RANTES expression) for clearance of infected cells.

**Fig. 2:**
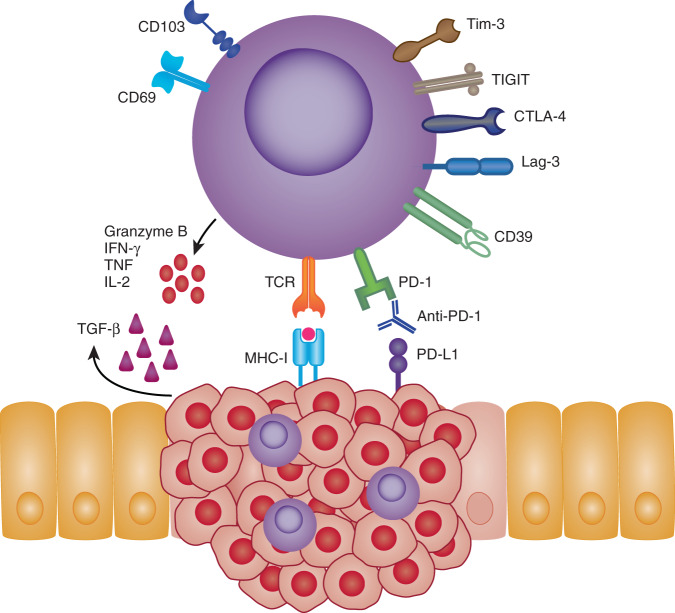
CD8 + TRMs in cancer. TRMs expressing CD103 and CD69 have been identified in numerous cancerous tissues. The presence of TRMs within and around tumor tissues are often associated with improved patient outcomes. Their favorable positioning within the tissues makes TRMs rapid first-line effectors that may suppress and eliminate local tumor growths. In an MHC-I dependent fashion, TRMs can directly lyse tumor cells via release of cytotoxic molecules, such as granzyme B and perforin. Alternatively, TRMs may also influence the local tumor microenvironment (TME) through their enhanced expression of inflammatory cytokines i.e., IFNγ, TNF and IL-2. Expression of inhibitory receptors such as PD-1, CTLA-4, LAG-3, TIM-3 and TIGIT have also found to be elevated on TRMs making them attractive targets for checkpoint blockade therapies. Indeed, tumor-specific TRMs targeted by anti-PD-1 show enhanced reactivity toward autologous tumor samples. Local signals within the TME such as TGF-b or VCAM-1 signaling may also shape TRMs phenotype and survival by influencing TRM markers such as CD103, thus improving retention within tumor tissues.

In this review we summarize our current knowledge of human TRM homeostasis and parallel their characteristics to studies derived from murine models. We focus in on four different mucosal sites—gastrointestinal (GI) tract, lung, head/neck and female reproductive tract (FRT) —and discuss the knowns and unknowns of TRM biology and differential functions in relation to intracellular pathogens (Fig. 1) and malignancies (Fig. 2).

### Human tissue-resident memory T cells (Trms)

Since the discovery of TRMs about a decade ago, multiple studies using parabiosis experiments, intravascular labeling, tissue grafting, and migration blockade have helped to elucidate the non-circulatory nature of memory T cells in several tissues of the mouse (reviewed in^17^). TRMs rapidly secrete cytokines and chemokines upon restimulation and offer increased protection from pathogen reinfections and tumor challenges, in comparison to re-circulating T cells^18,19^. Mechanistic demonstration of tissue residency is more difficult to prove in humans. As such, most human studies rely on using surface phenotypes and transcriptional signatures to designate resident lymphocytes. However, during the past years several innovative experiments have provided compelling evidence that TRMs most likely exist in human tissues. For instance, patients with cutaneous T cell lymphomas receiving alemtuzumab (anti-CD52 depleting antibody) experience complete depletion of recirculating TEM and TCM cells from blood and skin. Closer examination revealed that T cells with a conventional TRM phenotype (CD69 + CD62L−CCR7−) were largely unaffected multiple weeks after treatment^20,21^. Likewise, transplantation of HLA miss-matched organs, which allow the tracking of donor-derived TRMs from re-circulating recipient TCM and TEM cells, have demonstrated that donor-derived T cells, with a TRM phenotype, can be observed for 1–2 years after GI, lung and skin transplantation^22–24^. These studies particularly found that donor-derived CD69 + CD103 + TRMs were preserved for an extensive period, indicating that certain TRM populations may experience differential resident longevity. Pioneering studies by Farber et al. on human organ donors have further shown that virtually all organs have T cells with a TRM phenotype (CD69 + CCR7−) throughout a lifetime^25,26^. CD69 directly hinders T cells from migrating from tissues to blood through its binding to the egress receptor, S1PR1, on the cell surface^27^. This leads to the internalization and degradation of S1PR1, and the incapacity of T cells to respond to the S1P gradients in lymphatic vessels. Residency in humans is now, as such, usually inferred by high expression of CD69 and other retention markers and integrins (CD103 and CD49a), and low expression of egress markers (S1PR1, CD62L, and CCR7). The CD69 + CCR7− expression pattern is observed in a majority of human memory CD4 + and CD8 + T cells in SLTs and NLTs^25,26^, whereas memory T cells in the blood and lymph do not express CD69 at steady-state^28,29^. In early models, the protection afforded by TRMs was thought to primarily involve perforin-mediated transfer of serine proteases (granzymes), so called cytotoxic activity^13^. However, this paradigm does not necessarily apply to all TRMs across different tissue^24,28,30^, which instead exhibit a unique transcriptional profile compared to circulating cells^31^ and are thought to work partly as innate-like sensors^32^. As such, it is tempting to speculate that human TRMs might act through both cytotoxic mechanisms and recruitment of other immune subsets, to maintain full protection from pathogen invasion and malignancy development.

### Lung

The lung forms an important immunological barrier between the host and the external environment. Thus, TRMs act as important long-lived sentinels that protect against invading pathogens and survey tissues for malignant cells. As highlighted above, data from human lung transplants have shown that donor-specific CD4 + and CD8 + TRMs within transplanted tissue show up to 1 year maintenance within recipients^23^. Overtime, an accumulation of host-specific T cells can be seen within donor-tissue which acquire a TRM phenotype, suggesting de novo generation occurs via seeding of tissues by circulating T cells. Further, analysis of the TCR repertoire of CD4 + and CD8 + TRMs from human organ donors spanning over 50 years show stable TCR clonal maintenance suggesting these cells are constantly being replenished and maintained within the tissue as we age^33^. During homeostasis, both CD4 + and CD8 + TRMs within lung tissue display high baseline levels of mRNA encoding inflammatory markers such as IFNγ, granzyme B and TNF without need for stimulation^34,35^, emphasizing their role as fast acting mediators of immunity.

### Lung cancer

Current research on the role of TRMs in lung cancers have been focused on non-small-cell lung carcinoma (NSCLC), which accounts for 85% of all lung cancers in the US^36^. Infiltrating “TRM-like” CD4 + and CD8 + CD103 + cells have been detected within the TILs in NSCLCs, where studies have specifically demonstrated that CD8 + TRMs are associated with enhanced survival^37–41^. Although studies can transcriptionally confirm the phenotype of such T cells to have a TRM phenotype, i.e., through co-expression of CD69 or other inflammatory markers, it remains difficult in humans to delineate bona fide TRMs which arise during inflammation. This is because CD69 may be upregulated during TCR stimulation, hypoxia or type I interferon signaling and CD103 in presence of TGFb^42–45^. However, based on the fact that tumor-specific clonotypes are preferentially localized within the tumor microenvironment, and to a less degree in the peripheral blood, it seems likely that most CD103 + tumor-specific CD8 + T cells are TRMs. Within tumor tissue, lung CD8 + TRMs also display high levels of cytotoxic effector molecules, proliferation and express high levels of co-inhibitory markers PD-1, CTLA-4, LAG-3 and Tim-3^37,39,46^, making them attractive targets for checkpoint inhibitor therapy. Indeed, CD8 and CD103 co-expression has been shown to be a predictive marker in positive response to anti-PD-L1 treatment in NSCLC^47^. Moreover, ex vivo stimulation of lung-resident CD8 + TRMs with anti-PD-1 enhances their IFNγ secretion and their capacity to kill autologous tumor cells^37,46^. Thus, accumulating evidence suggest that TRMs contribute to tumor control in NSCLC (Fig. 2).

### Influenza A virus (IAV)

IAV is a highly contagious virus which accounted for an estimated 9.5 million hospitalizations in 2017^48^, and its consequent respiratory disease, the ‘flu’, has significant mortality rates among immunocompromised individuals. Several human studies have described the detection of CD4 + and CD8 + TRMs within lung tissue after infection with IAV^49–53^. Ex vivo stimulation of IAV-specific CD8 + TRMs results in proliferation and polyfunctional inflammatory cytokine production, including IFNγ, TNF, and IL-2^54^ (Fig. 1). Subsequent re-infection of individuals with seasonal variations of IAV has posed the question whether TRMs offer heterosubtypic protection after seeding the lung tissue. Indeed, both murine and human studies indicate that CD4 + and CD8 + TRMs play a significant role in heterosubtypic immunity providing protection upon secondary infection^55,56^. Interestingly, unlike their other mucosal counterparts, murine studies have also shown that IAV-specific CD8 + TRM numbers appear to diminish overtime, thought to be in part contributed by increased apoptosis^57^. Recently, this phenomenon has been also described in human lung tissue spanning 22–68 years of age; compared to other T cell subpopulations, CD8 + TRMs show the most drastic decline in cell numbers associated with age^58^. Moreover, in vitro cultures of lung tissue from these tissues with IAV have revealed a reduced capacity of CD8 + TRMs to produce IFNγ with age^58^. There data indicate that the loss of functional CD8 + IAV-specific TRMs overtime may contribute to diminished long-term heterosubtypic immunity in elderly indivduals.

### Coronaviruses

Severe acute respiratory syndrome coronavirus (SARS-CoV), middle east respiratory syndrome coronavirus (MERS-CoV) and severe acute respiratory syndrome coronavirus 2 (SARS-CoV-2) represent different examples of severe coronaviruses that have emerged during the past two decades from zoonotic sources. All three viruses cause acute respiratory illnesses as they infect lung cells, damaging airways and alveolar epithelial cells^59^. Despite the extraordinary efforts recently made into Covid-19 research (disease caused by SARS-CoV-2), it remains unknown whether TRMs can attenuate Covid-19 severity in humans^60^. Studies on MERS-CoV and SARS-CoV, have reported a clear role of respiratory T cells for the protection against severe disease in the lung^59,61^. There is now also evidence that SARS-CoV-2-specific CD4 + and CD8 + T cells with TRM characteristics are found in lung lavages from convalescent patients^62^. Likewise, a new study recently demonstrated that pre-existing SARS-CoV-2-reactive CD8 + TRMs are present in tonsils from a large proportion of unexposed individuals^63^. As such, cross-reactive TRMs might contribute to rapid sentinel responses to SARS-CoV-2 in newly exposed donors. Analyses of vaccine-induced TRMs have been rare due to limited access to patient samples, however intranasal delivery of a ChAd-SARS-CoV-2-S vaccine in mice incudes localized CD8 + TRM responses, which correlate with reduced viral replication and shedding in comparison to systemic delivery^64^. As such, it remains unclear if intramuscular injection with the current Covid-19 vaccines will induce local cell-mediated immunity, including CD4 + and CD8 + TRM responses, in the respiratory tract.

### Tuberculosis

Tuberculosis (TB) is one of the leading causes of death worldwide and is caused by the infectious agent (Mycobacterium tuberculosis, Mtb). Although many studies have demonstrated on the importance of T cells to protect from active TB infection, the major obstacle in development of an effective vaccine is a lack of defined immune correlates. Nevertheless, numerous murine studies have deciphered the immune responses generated by the only licensed TB vaccine: Mycobacterium bovis-derived Bacillus Calmete Guérin (BCG)^65^. BCG protects infants, but demonstrate poor protection of pulmonary TB in adolescents and adults^66^. Studies have shown that blockade of lymphocyte tissues egress, by FTY720 administration, after BCG vaccination does not negatively impact outcome after TB challenge^67,68^, indicating that lung CD4 + and/or CD8 + TRMs are sufficient for protection. The accumulating body of evidence that TRMs may mediate protection to TB have guided current vaccine efforts to generate TRMs via vaccination. Through various strategies such as prime-boost, adjuvanted and viral vectors of Mtb antigens and/or BCG, several groups have been able to effectively generate both CD4 + and CD8 + lung TRMs that confer enhanced protection against TB challenge^69–73^. Similar induction of lung CD4 + and CD8 + TRM have been obtained in studies with non-human primates using BCG or subunit vaccines delivered intravenously or intranasally^74–78^. In one of these recent studies^78^, the authors found that intravenous immunization with BCG induced high levels of CD4 + and CD8 + TRMs across the entire lung parenchyma, where nine of ten animals demonstrated durable protection from Mtb infection. Collectively, vaccine studies in murine and non-human primates suggest that induction of robust CD4 + and CD8 + TRM responses through specific routes might become a rationale to induce an effective vaccine response against TB.

### Gastrointenstinal (GI) Tract

The GI tract form an essential interface to the external environment and maintain constant balance between protection against invading pathogens and tolerance of nonpathogenic bacteria and food antigens. The GI tract consists of the esophagus, stomach, pancreas, liver, and intestinal tissues. In humans, all these tissues have been reported to harbor a large proportion of CD4 + and CD8 + TRMs^25,79–81^. However, for the purposes of this review, we will focus on the lower GI tract, namely the gastric and intestinal tissues which contain a diverse population of immune cells where a large proportion of the CD4 + and CD8 + T cell compartment (up to 90%) display phenotypic features of TRMs^31,82^. These cells seed the intestinal mucosa and in contrast to other T cell subsets, are stably maintained over a lifetime^24,26,83,84^. Studies from both human intestinal allografts and non-human primate allogeneic hematopoietic stem cell transplants demonstrate that circulating CD4 + and CD8 + T cells may enter the intestinal mucosa and acquire a TRM phenotype^85,86^, which is thought to be largely contributed by constitutive expression of TGF-b within the intestinal epithelium that drives CD103 expression^87–90^.

### Gastric adenocarcinoma

Gastric adenocarcinoma, which makes up 95% of all gastric cancers, is the fifth most diagnosed and third most lethal cancer worldwide^91^. Several recent studies have shown that CD8 + TRMs readily infiltrate gastric cancers, produce high levels of inflammatory cytokines and are associated with better prognosis^92–94^. One in vitro study showed that CD8 + CD103 + T cells produce higher levels of IFNγ and TNF when co-cultured with tumor cells and had higher expression of PD-1, TIGIT and CD39 in comparison to CD103- T cells^92^. Survival of CD8 + TRMs was dependent on fatty-acid metabolism where tumor cells outcompeted CD8 + TRMs leading to cell death. However, co-culture with anti-PD-L1 reverse this phenotype, resulting in enhanced fatty-acid uptake in CD8 + TRMs and enhanced survival, again demonstrating TRMs as an attractive target for checkpoint blockade therapies. Indeed, subsequent studies have shown that patients with high infiltrating CD103 + CD8 + T cells receiving anti-PD-1 have higher overall survival compared to total CD103- CD8 + T cell and were highly associate with tertiary lymphoid structures^93,94^. Collectively, these data suggest that CD8 + TRMs might be attractive targets for future immunotherapies in gastric cancer.

### Colorectal cancer

Colorectal cancer (CRC) ranks as the 3rd leading cause of cancer-related deaths worldwide^95^. Colorectal cancers (CRCs) can be roughly divided based on presence or deficiency of the patient’s mismatch repair system (MMR). Around 10–20% of patients with CRC will be deficient for MMR^96,97^ and these patients have a higher survival rate which is thought to be contributed by a higher mutational burden of up to 100-fold^98,99^, leading to enhanced immune infiltration within the tumor^100,101^. Few human studies have investigated the role of TRMs within CRCs, however, recently CD8 + TRMs were shown to be enriched within MMR-deficient CRC tissues compared to healthy mucosa or MMR-stable CRC tissues^102^. Treatment with anti-PD-1 therapy improves survival in patients with MMR^101^, and CD4 + and CD8 + TRM-like cells with high expression of PD-1 can be detected within MMR-deficient CRC tissues^102^. No study has yet evaluated whether TRMs within CRC are the cells that are reactive to anti-PD-1 therapy and convey protection, however, their reactivity within other cancer types including gastric cancer, highly suggest that these cells play a key role in checkpoint blockade therapy in CRC (Fig. 2).

### HIV

The human immunodeficiency virus (HIV) infects primarily memory CD4 + T cells, leading to a diminished capacity of the immune system to defend against otherwise relatively harmless infections. Without antiretroviral therapy (ART), most humans eventually progress to acquired immunodeficiency syndrome (AIDS). Although most HIV studies have been conducted on peripheral blood, new data on non-human primate studies suggest that the GI tract harbors 98% of the total viral reservoirs during ART^103^. This is most likely a consequence of the early seeding of CCR5 + CD4 + T cells within the gut with viral particles^104^. Whether CD4 + TRMs represent a cellular reservoir, similar to cervix^105^, remains unknown. Similar to other studies in humans, it is hard to infer phenotypes to a bona fide TRM status in HIV-infected tissues. Nevertheless, previous studies have found that HIV-specific T cells are generally present at higher magnitudes in SLTs and the GI tract compared to blood; despite the fact that most HIV-specific T cells lack homing receptors to lymphoid^106–108^. In addition, most HIV-specific CD8 + TRMs express CD69 and intermediate levels of CD103 in rectosigmoid and demonstrate polyfunctional characteristics^109^ (Fig. 1). HIV-specific CD8 + TRM responses are strongest in individuals naturally controlling HIV, suggesting that CD8 + TRMs mediate protection against HIV disease progression^28^. Recent vaccine studies in non-human primates further suggest that induction of CD8 + TRM responses may lower the threshold of neutralizing antibodies to confer durable protection after lentiviral challenge^110^. Overall, these data indicate that future HIV vaccine and cure regimens might benefit from inducing local TRM responses.

### Female reproductive tract (FRT)

The mucosal immune system in the FRT is an early barrier against pathogenic organisms, including sexually transmitted diseases of epidemic proportions (e.g., Chlamydia, Herpes Simplex virus (HSV) or HIV)^111^. Two types of FRT mucosal surfaces exist: upper FRT (endometrium and endocervix) lined with a type I single columnal epithelial layer and lower FRT (vagina and ectocervix), with a type II lining compromised of several stratified squamous epithelial layers linked to a basement membrane. Mucosa-associated lymphoid tissues (MALT) are only present in the upper FRT, where T cell infiltration mainly occurs under inflammatory conditions^112^. Numerous murine studies have demonstrated that long-lived CD8 + TRMs can be established during infection and generated through vaccination in the FRT and can protect against infectious pathogens such as HSV^113–119^. Emerging data in the human field suggest that CD8 + TRMs expressing higher levels of CD103 and cytotoxic molecules (e.g., Granzyme B) are close to the epithelium, whereas lower levels of CD103 are present on CD8 + TRMs in the stroma^120^, indicating that distinct antigen-specific CD8 + TRM subsets are positioned throughout the human cervix.

### Cervical cancer

Cervical cancer is the leading cause of cancer-related deaths in women, where up to 75% of cervical cancers are caused by HPV16 and HPV18 infection^121^. Limited human studies exist investigating the role of CD4 + and CD8 + TRMs within cervical cancers. Nevertheless a recent study found favorable prognosis in individuals with high infiltration of CD8 + CD103 + T cells^122^. These CD8 + TRM-like cells also expressed high levels of granzyme B and PD-1^122^. In the same study, irradiation combined with an E6/7 targeting vaccine delivered therapeutically was able to induce infiltrating HPV-specific CD8 + TRMs within a preclinical cervical cancer model. An earlier preclinical study also showed that intravaginal vaccination with an HPV vector could induce cervicovaginal localized CD8 + TRMs^115^. However, clinical trials with therapeutic vaccination against HPV-induced cervical cancers have so far yielded poor results, indicating that more work is required to address whether CD8 + TRMs can be harnessed for HPV-induced cervical cancer therapies.

### Herpes simplex virus 2

HSV-2 infection is almost exclusively sexually transmitted and the main cause of genital herpes. The virus leaves most often no symptoms and remains lifelong, latent and incurable. Numerous studies in murine models have shown that HSV-specific CD8 + TRMs can be generated within the genital mucosa and surrounding tissue^115–119^. Despite not containing MALT in the steady state, both human CD4 + and CD8 + TRMs can be established in the lower FRT after intravaginal infections, and low numbers of those persist as TRMs for several months after lesion healing^123^. More specifically, they are located in the dermo-epidermal junction and provide immune surveillance along the nerve endings^124^. Thus, the severity of HSV-2 infection may depend on the CD4 + and CD8 + TRM density in the specific infection site^125^, where TRMs mainly rely on cytokine secretion (mostly IFNγ), rather than cytotoxicity effect for viral clearance^126,127^ (Fig. 1). As such, these data suggest that CD4 + and CD8 + TRM subsets in the cervix provide immune surveillance mechanisms to control HSV-2 replication^120^.

### Head and neck

The upper aerodigestive tract consists of several distinct anatomical sites lined by mucosal epithelia including paranasal sinuses, nasal and oral cavities, pharynx, and larynx. Populations of CD4 + and CD8 + TRM can be readily detected in associated oropharyngeal lymphoid structures such as the tonsils. Moreover, studies in Epstein-Barr virus (EBV) infection show a preferential accumulation of antigen-specific CD8 + TRM within the tonsils during and after infection suggesting an essential role in control and protection against upper-respiratory tract pathogens^25,128–130^.

### Head and neck cancer

Head and neck squamous cell carcinomas (HNSC) encompass several malignancies that originate in the mucosal lining of the upper aerodigestive tract. It is now widely accepted that the major risk factors for these diseases are smoking tobacco and alcohol use^131–133^. Furthermore, high-risk human papilloma virus (HPV) infection is another major risk factor, where HPV-16 strain accounts for over 90% of HPV-related HNSC. Vaccination against HPV has led to a substantial decrease in the incidence of cervical cancer across a number of countries and is known to be dependent on the formation of neutralizing antibodies against the virus^134^. Whether HPV vaccination can induce protection against HPV-related HNSC and if CD4 + and CD8 + TRMs may play a role, has yet to be fully studied. However, a recent murine study using mucosal vaccination with E7 protein from HPV-16 has shown that CD8 + TRM can be induced within the head and neck of mice which correlate with protection against orthotopic tumor challenge^40^. It is also evident from clinical studies that CD8 + TRM play a key role in immunity against HNSC as infiltration of the tumor with CD103 + TILs are a prognostic marker for enhanced survival across several HNSCs and these cells are able to kill autologous tumor cells in an MHC-I dependent manner^44,135,136^. Although studies have largely focus on CD8 + TRM within HNSC, interestingly, the tumor microenvironment itself may play a role in inducing and sustaining both CD4 + and CD8 + TRM populations within the tumor. A recent study has shown that mesenchymal stromal cells that accumulate within HSNC also provide survival signals and induce expression of CD4 + and CD8 + TRM surface markers through VCAM1^137^.

### CMV

CMV persists as a latent infection and can reactivate to lytic infection at various mucosal tissues, including tonsils and salivary glands. While most individuals are asymptomatic, immunocompromised patients are more susceptible for CMV morbidity and mortality^138^. CMV-specific T cells are critical to control CMV reactivation, as evidenced in clinical settings using infusion of CMV-specific T cells to prevent organ dysfunction^139^. Furthermore, immune-deficient patients that experience uncontrolled viral replication and end-organ disease usually have impaired T cell responses^140,141^. Murine CMV models (MCMV) have identified MCMV-specific CD4 + and CD8 + T cells with a TRM phenotype in the salivary glands^142^. Most our knowledge of human CMV-specific T cell immunity is limited to peripheral blood samples, where these cells generally harbor a unique terminally-differentiated phenotype with high cytotoxic activity^10^. Studies derived from tissues have instead shown that CMV-specific T cells generally possess a recirculating TEM phenotype, enabling them to continuously migrate into mucosal tissues^143^. Recent studies using human organ donors have identified the lung as a large CMV reservoir, where CD8 + T cells are often activated, whereas tonsil and salivary glands seem to serve as long-term reservoirs of the CMV-specific T cells^143^. Most commonly CMV-specific CD8 + T cells display variable levels of CD69 and low CD103 expression in tonsils and SLTs, instead suggesting that these cells might preferentially rely on re-circulation mechanisms to provide protection against CMV reactivation^28,144^.

### EBV

EBV infects more than a 90% of the worldwide population. It is usually acquired asymptomatically in childhood, establishing a latent infection of the memory B cell pool, but when acquired through saliva in adolescence, it can cause Infectious Mononucleosis disease (IM)^145^. However, many of the B cells downregulate antigen expression, becoming part of a latent pool of infected antigen-negative genome-positive memory B cells, which recirculates between the tonsil (pharyngeal lymphoid tissues) and blood^146^. The expression of both lytic and latent proteins in primary infection is immunogenic and triggers the recruitment of EBV-specific CD8 + TRMs to the tonsil. Many EBV-specific CD8 + T cells express both CD69 and CD103, and are positioned along the epithelial barrier, which could explain why EBV reactivation is often asymptomatic or sub-clinical^144^ (Fig. 1). A recent study from our group confirmed these results in human tonsils and additionally showed higher frequencies of EBV-specific CD8 + T cells in tonsils than blood^63^—probably a direct consequence of their residency status. Moreover, this study indicated that EBV-specific CD8 + TRMs are polyfunctional and express CXCR5, to directly spearhead the antiviral response against the virus in B cell follicles^63^.

## Conclusions

The identification of TRMs have caused a paradigm shift in our understanding of T cell-mediated immunity. We know believe that TRMs, and not only re-circulating T cells, underpins immune surveillance of tissues to mediate proper protection and control of infections and malignancies. Despite the explosion of studies centered on human TRM responses, there is still a general lack of knowledge of relatively trivial pathogen-specific TRM characteristics (e.g., phenotype and functions) in contrast to blood; and although it is clear that certain TRM populations exist between mucosal sites, we still do not know if distinct TRM populations could impart differential immune pressure on tumors and pathogens within and between different sites. A further, and ultimate, goal in the future will be to translate our knowledge derived from TRM studies in mice to humans and develop new types of vaccines through mucosal routes that can induce long-lived TRM responses. This might be a future path forward, for instance in Covid-19 vaccine development, given the recent surge in variants of concern. Continual studies are as such needed to integrate mouse and human studies to harness the full potential of TRMs at mucosal surfaces.
